# Emerging trends and focus of human gastrointestinal microbiome research from 2010–2021: a visualized study

**DOI:** 10.1186/s12967-021-03009-8

**Published:** 2021-07-31

**Authors:** Xingzhu Yuan, Chengting Chang, Xinrong Chen, Ka Li

**Affiliations:** West China School of Nursing/ West China Hospital, Sichuan University, No.8 Teaching Building, Chengdu City, 610041 Sichuan province China

**Keywords:** Gastrointestinal microbiome, Human, Bibliometric, Visualized study, Scopus, Emerging trends, Research focus

## Abstract

**Background:**

The gastrointestinal microbiome is an important component of the human body and is closely related to human health and disease. This study describes the hotspots of the human gastrointestinal microbiome research and its evolution in the past decade, evaluates the scientific cooperation network, and finally predicts the field’s future development trend using bibliometric analysis and a visualized study.

**Methods:**

We searched the original articles from January 2010 to February 2021 in the Scopus database using the term “gastrointestinal microbiome” and its synonyms. CiteSpace was used to construct country and author co-occurrence map; conduct journal, citation cocitation analysis, and reference co-citation knowledge map; and form a keywords co-occurrence map, a clustering knowledge map, timeline view of keywords, and burst term map.

**Result:**

A total of 4444 documents published from January 2010 to February 2021 were analysed. In approximately the past decade, the number of articles on the human gastrointestinal microbiome has increased rapidly, and the research topics focus on different populations, research methods, and detection methods. All countries and regions in the world, led by the US, are studying the human gastrointestinal microbiome, and many research teams with close cooperation have been formed. The research has been published extensively in microbiology journals and clinical medicine journals, and the highly cited articles mainly describe the relationship between gastrointestinal microorganisms and human health and disease. Regarding the research emphasis, researchers' exploration of the human gastrointestinal microbiome (2011–2013) was at a relatively macro and superficial stage and sought to determine how the gastrointestinal microbiome relates to humans. From 2014 to 2017, increasingly more studies were conducted to determine the interaction between human gastrointestinal flora and various organs and systems. In addition, researchers (2018–2021) focused on the gastrointestinal microbial community and the diversity of certain types of microbes.

**Conclusion:**

Over time, the scope of the research on the clinical uses of the gastrointestinal microbiome gradually increased, and the contents were gradually deepened and developed towards a more precise level. The study of the human gastrointestinal microbiome is an ongoing research hotspot and contributes to human health.

## Background/introduction

It is currently well appreciated that diverse microbial communities reside within the intestinal tract, on the skin, and on nearly all of the exposed surfaces of the human body [1]. The human gastrointestinal (GI) tract harbours the highest density and complexity of microbial organisms in the body [2], and the gastrointestinal microbiota has a level of complexity comparable to that of an organ system [3]. A key role of the gastrointestinal microbiome in the establishment and maintenance of health, as well as in the pathogenesis of diseases, has been identified over the past two decades [4]. In addition, the relationship between the gastrointestinal microbiome and populations with different ages and genders has been gradually revealed [5, 6]. An increasing number of gastrointestinal microbiome detection methods, such as Polymerase Chain Reaction (PCR) or Fluorescence in situ hybridization (FISH), have also emerged [7, 8]. Importantly, the microbiome (including bacteria, viruses, fungi, etc.) regulates health, and its alterations can contribute to disease [9]. A large number of systematic reviews and meta-analyses have shown that gastrointestinal microorganisms are interrelated with inflammatory bowel disease (IBD), irritable bowel syndrome (IBS), diabetes, hepatitis, and autism in humans [10–14]. For example, IBS can disturb the intestinal microecology, which may continue to aggravate IBS. Conversely, the improvement of the intestinal microecology using probiotics and other means may be conducive to the alleviation of the symptoms of IBS [15–17]. However, most research on the gastrointestinal microbiome is still in the stage of animal experiments, and the results of animal studies cannot be directly applied to humans. For instance, bidirectional microbiota-gut-brain communication has mostly been explored in animal models with human research lagging [18].

Characterizing the structure of knowledge, the evolution of research topics, and the emergence of topics have always been an important part of information science (IS) [19]. Bibliometric analysis is an important tool in assessing the research activity and research trends on a particular topic, as well as the most prominent research trends, for future research. A knowledge map, visualizing the connections between complex silos of information, is one way to accurately capture and display disparate pieces of information [20]. Moreover, key researchers, countries, and collaboration networks between leading research groups can be identified [21, 22]. However, previous bibliometric analysis or visualized study of the gastrointestinal microbiome did not exclude animal experiments, and the articles related to the human gastrointestinal microbiome were not analyzed separately. Such research could not describe how well the gastrointestinal microbiome works in humans. Therefore, it is necessary to conduct systematic, intuitive, and scientific bibliometric analysis and visualized study of the growing number of original research articles on the human gastrointestinal microbiome.

This study aims to visualize articles on the human gastrointestinal microbiome in the last ten years by using knowledge maps. We described the research hotspots of the human gastrointestinal microbiome and its evolution in the past decade, evaluated the scientific cooperation network, discussed the relationship between humans and gastrointestinal microbiomes, and predicted the field’s future development trend.

## Methods

The literature data used in this study were downloaded from the Scopus database, which is widely accepted among researchers conducting high-quality bibliometric analyses [23–26]. We used “gastrointestinal microbiome” for topical retrieval and the following search queries in titles, abstracts, and keywords: (gastrointestinal AND microbiome), (gut AND microbiota), (gut AND flora), (intestinal AND microbial AND population), (intestinal AND microecology), (enteric AND microorganism), (gut AND microecology), and (intestinal AND microorganism). In addition, the time was defined as “2010–2021” without any language limitation. The above keywords were chosen from a list of Medical Subject Headings (MeSHs) provided by the National Library of Medicine (NLM)/PubMed. The literature type was defined as “articles”. Studies in the subject areas of veterinary, poultry science, soil biology, dentistry, engineering, material science, animal experiments, in vitro cell culture experiments, and secondary studies were excluded.

CiteSpace (Chaomei Chen, China), a freely available software tool for analysis, was used to make visualization maps in this study. Developed by Chaomei Chen in 2004 at Drexel University (USA), CiteSpace is usually used to analyze, detect and visualize trends and patterns in scientific literature [27]. The principle of the software includes coword analysis used to measure the number of occurrences of a group of words (keywords, authors, regions, and citations) in the same group of literature and to perform matrix analysis [28]. In this paper, we use CiteSpace 5.5.R2 to construct country and author co-occurrence map; conduct journal, citation cocitation analysis, and reference co-citation knowledge map; and form a keywords co-occurrence map, a clustering knowledge map, timeline view of keywords, and burst term map.

## Results

### Distribution of articles by publication years

**Fig. 1:**
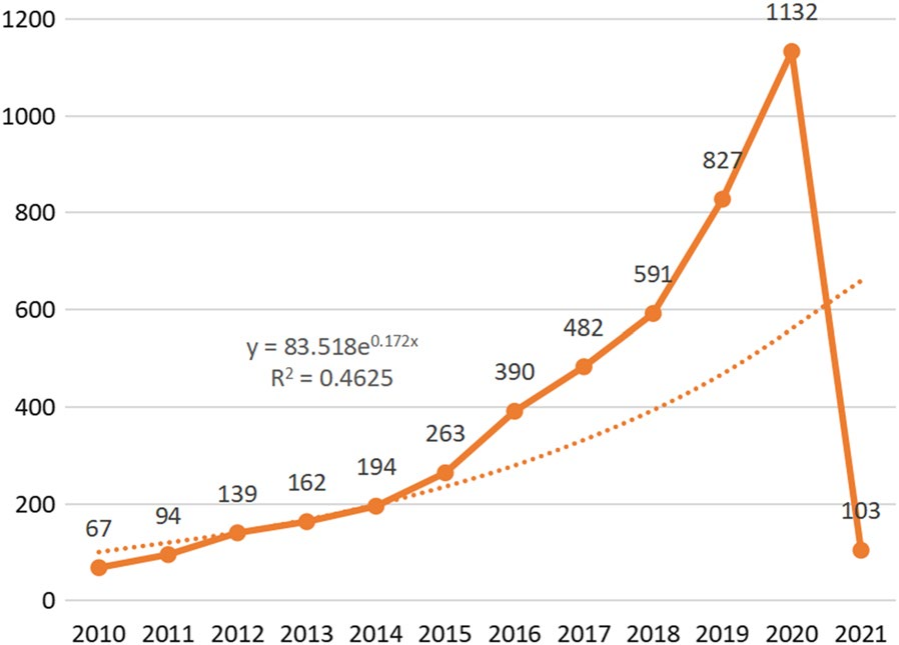
Time-trend distribution of articles in the field of human gastrointestinal microbiome

Overall, 4444 documents published from January 2010 to February 2021 were analysed. The number of annual documents during this period showed an exponential growth trend (y = 83.518e0.172x, R2 = 0.4625). The specific numbers of annual documents are shown in Fig. 1.

### Scientific cooperation network analysis

**Fig. 2:**
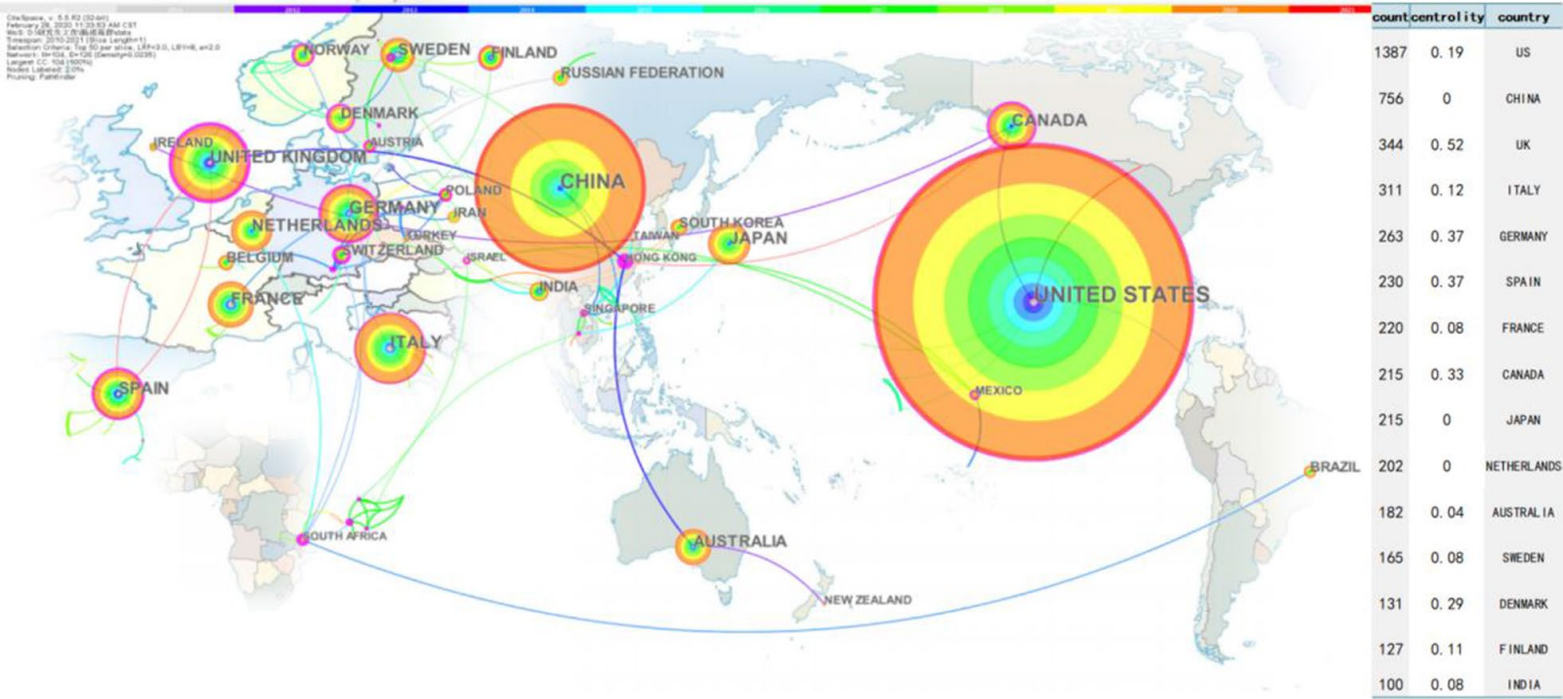
The country co-occurrence knowledge map of the human gastrointestinal microbiome during 2010–2021. Nodes show in the form of annual rings that the annual ring width represents how many papers the country/region publishes in a given year. The more papers are published, the wider the annual ring is in that year [28]

**Fig. 3:**
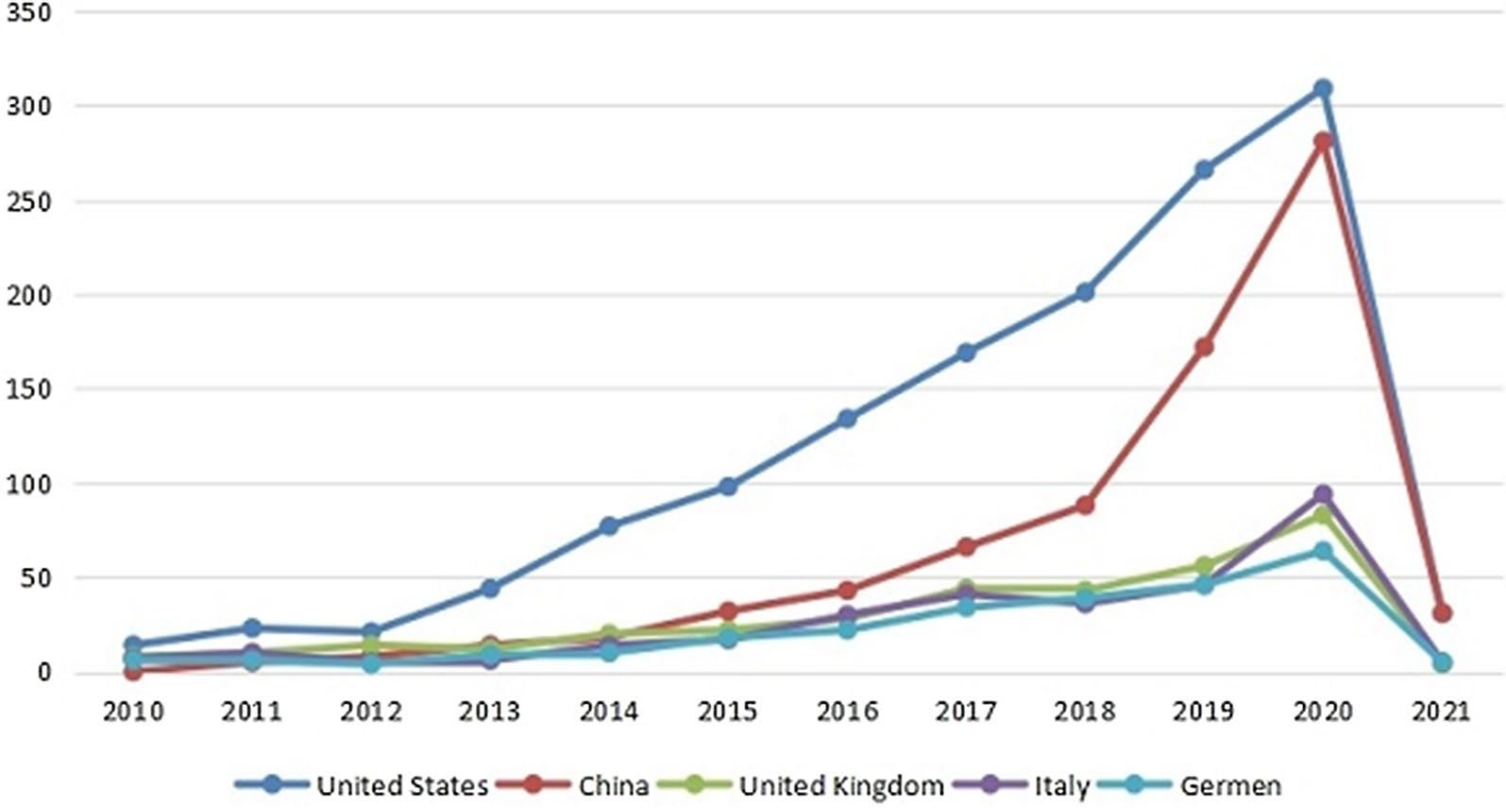
Annual distribution trends in the top five most published countries in the field of human gastrointestinal microbiome

In the country co-occurrence knowledge map (Fig. 2), 4444 articles about the human gastrointestinal microbiome were published by research groups in 104 countries or regions. There are 105 nodes and 128 lines, and the centrality is 0.02. The landmark node includes the United States with a count of 1387, China with a count of 756, the United Kingdom with a count of 344, Italy with a count of 311, and Germany (263 texts). The turning points with more connections include Hong Kong, South Africa, the United Kingdom, Switzerland, and Germany. The annual distribution trends in the top five most published countries was shown in Fig. 3. The United States has been leading the way in annual publication.

**Fig. 4:**
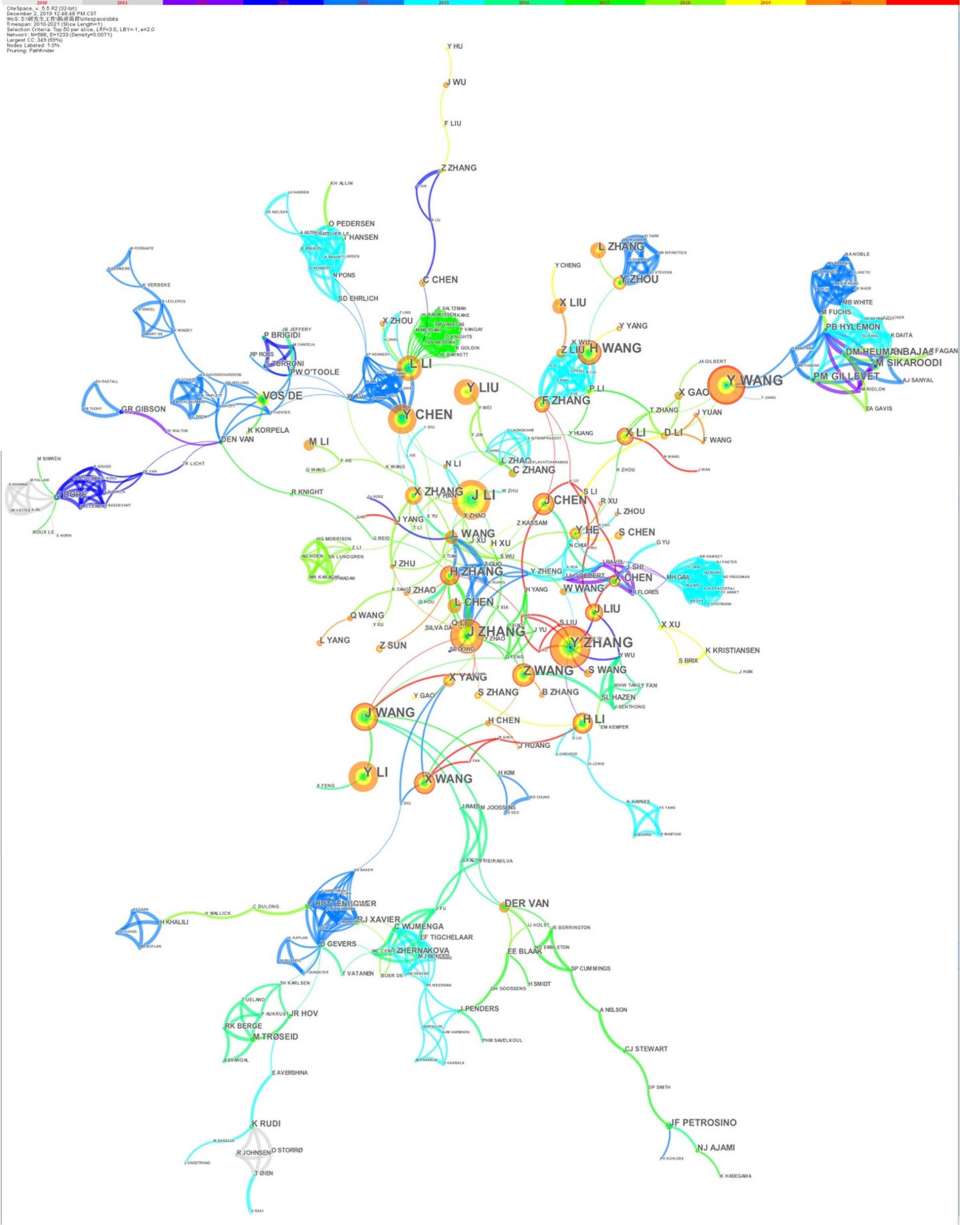
The author co-occurrence knowledge map of the human gastrointestinal microbiome during 2010–2021. The connection between nodes represents the cooperation between authors, and the width of the connection represents the strength of cooperation. The color of the connection represents the author’s first cooperation time. After the network is generated, the author’s cooperation will form several natural clusters. The author cooperation within the cluster is close, and the author cooperation between each class is less [28]

In the author’s co-occurrence knowledge map (Fig. 4), there are 588 nodes and 1233 lines, and the centrality is 0.007. The landmark nodes include Y Zhang, Y Wang, J Li, J Zhang, and Y Chen. The turning points with more connections include L Wang, L Li, X Yang, Y Chen, and J Wang.

### Journal and citation analysis

**Fig. 5:**
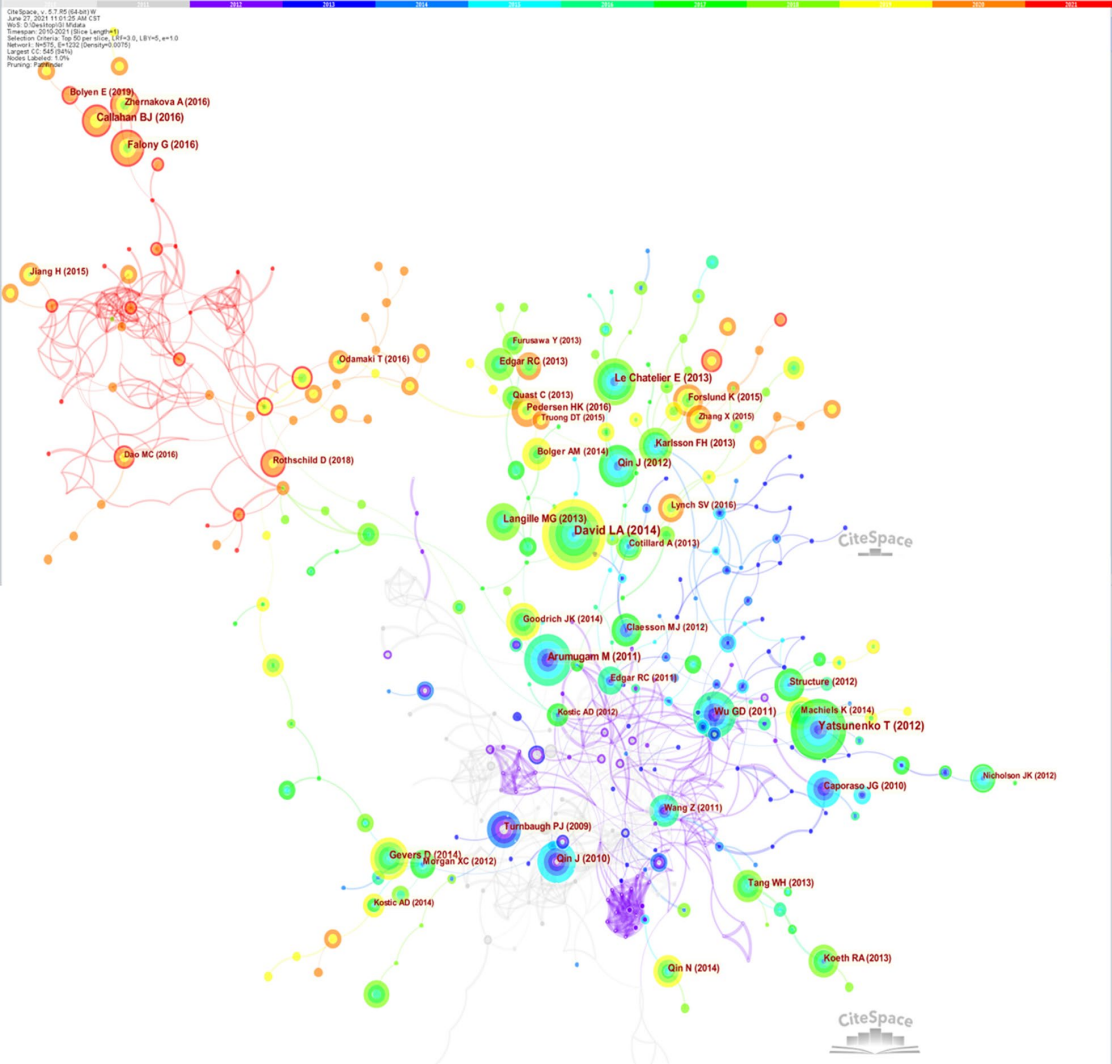
The co-citation knowledge map of the human gastrointestinal microbiome during 2010–2021

A total of 1255 journals published the 4444 articles on the human gastrointestinal microbiome. We analyzed the top ten journals that published articles on the human gastrointestinal microbiome (Table 1), reference co-citation knowledge map (Fig. 5) and the details of the top ten articles with the most citations on the human gastrointestinal microbiome in the last ten years (Table 2).

**Table 1:** Top 10 journals that published articles on human gastrointestinal microbiome during 2010–2021

Journal	Frequency	JC	IF	Country	Main ideas
Scientific Reports	326	Q1	3.999	UK	The natural and clinical sciences
Frontiers in Microbiology	91	Q2	4.237	Switzerland	The entire spectrum of microbiology
Microbiome	86	Q1	11.606	UK	The study of microbial communities, such as, microbial surveys, bioinformatics, meta-omics approaches and community/host interaction modeling
Microorganisms	78	Q2	4.151	Switzerland	Microbial physiology, Microbial ecology, Microbial genetics, Evolutionary microbiology, Systems microbiology, Medical microbiology and so on
Gut Microbes	70	Q1	7.744	US	Cutting-edge research on all aspects of microorganisms populating the intestine
Gut	54	Q1	19.818	UK	Clinical research of the alimentary tract, the liver, biliary tree and pancreas
Journal of pediatric gastroenterology and nutrition	46	Q1	2.938	US	Normal and abnormal functions of the alimentary tract and its associated organs and emphasis on development and its relation to infant and childhood nutrition
Frontiers in Cellular and Infection Microbiology	42	Q2	4.122	Switzerland	All areas of pathogenic microorganisms and their interaction with the hosts
Beneficial Microbes	40	Q2	3.374	Netherlands	The promotion of the science of microbes beneficial to the health and wellbeing of man and animal
BMJ Open	40	Q2	2.498	UK	Medical research from all disciplines and therapeutic areas

**Table 2:** The top 10 co-cited articles of the human gastrointestinal microbiome during 2010–2021

Author	Year	Cited by		Title	Journal	IF(2020)	Term	Methods
		Human Gastrointestinal Microbiome articles	wos					
Tanya [29]	2012	245	3575	Human gut microbiome viewed across age and geography	Nature	42.778	Gut microbiomes differ among human populations	16S rRNA
Lawrence [30]	2014	198	3967	Diet rapidly and reproducibly alters the human gut microbiome	Nature	42.778	Diet	16S rRNA
Qin [31]	2012	179	2909	A metagenome-wide association study of gut microbiota in type 2 diabetes	Nature	42.778	Type 2 diabetes	MGWAS analysis
Manimozhiyan [32]	2011	173	3384	Enterotypes of the human gut microbiome	Nature	42.778	Enterotypes	Metagenomes
Wu [33]	2011	166	3091	Linking long-term dietary patterns with gut microbial enterotypes	Nature	42.778	Diet	16S rDNA
Emmanuelle [34]	2013	155	1973	Richness of human gut microbiome correlates with metabolic markers	Nature	42.778	Metabolic markers and obesity	Quantitative metagenomic
Qin [35]	2010	144	5551	A human gut microbial gene catalogue established by metagenomic sequencing	Nature	42.778	Human gut microbial gene catalogue	Metagenomes
Morgan [36]	2013	120	4123	Predictive functional profiling of microbial communities using 16S rRNA marker gene sequences	Nature biotechnology	36.558	A computational approach to predict the functional composition of a metagenome using marker gene data and a database of reference genomes	PICRUSt
Benjamin [37]	2016	76	3974	DADA2: High-resolution sample inference from Illumina amplicon data	Nature Methods	30.820	The software package DADA2 for modeling and correcting Illumina-sequenced amplicon errors	DADA2
Gwen [38]	2016	57	766	Population-level analysis of gut microbiome variation	Science	41.847	Fecal microbiome variation in the average, healthy population	16S rRNA

*16S rRNA* 16S ribosomal RNA, *MGWAS* *analysis* metabolome-based genome-wide association studies

Six of the top 10 journals specialize in microbiology: two are general medicine journals, and the rest are gut and nutrition journals. Most of these journals are of good quality and are European and American journals.

An overview of the 427 top-cited articles among the 1,13,598 articles in the human gastrointestinal microbiome literature and the details of the ten most cited articles on the human gastrointestinal microbiome are summarized in Table 2. The size of a node represents the number of times the corresponding article has been cited in the dataset.

The highly cited articles on the human gastrointestinal microbiome have been published in top journals such as Nature and Science. The topics of these literatures include the relationship between the human gastrointestinal microbiome and diet, diabetes, human health and metabolism, and microbiome analysis methods.

### Emerging trends and research focus based on keywords analysis

**Fig. 6:**
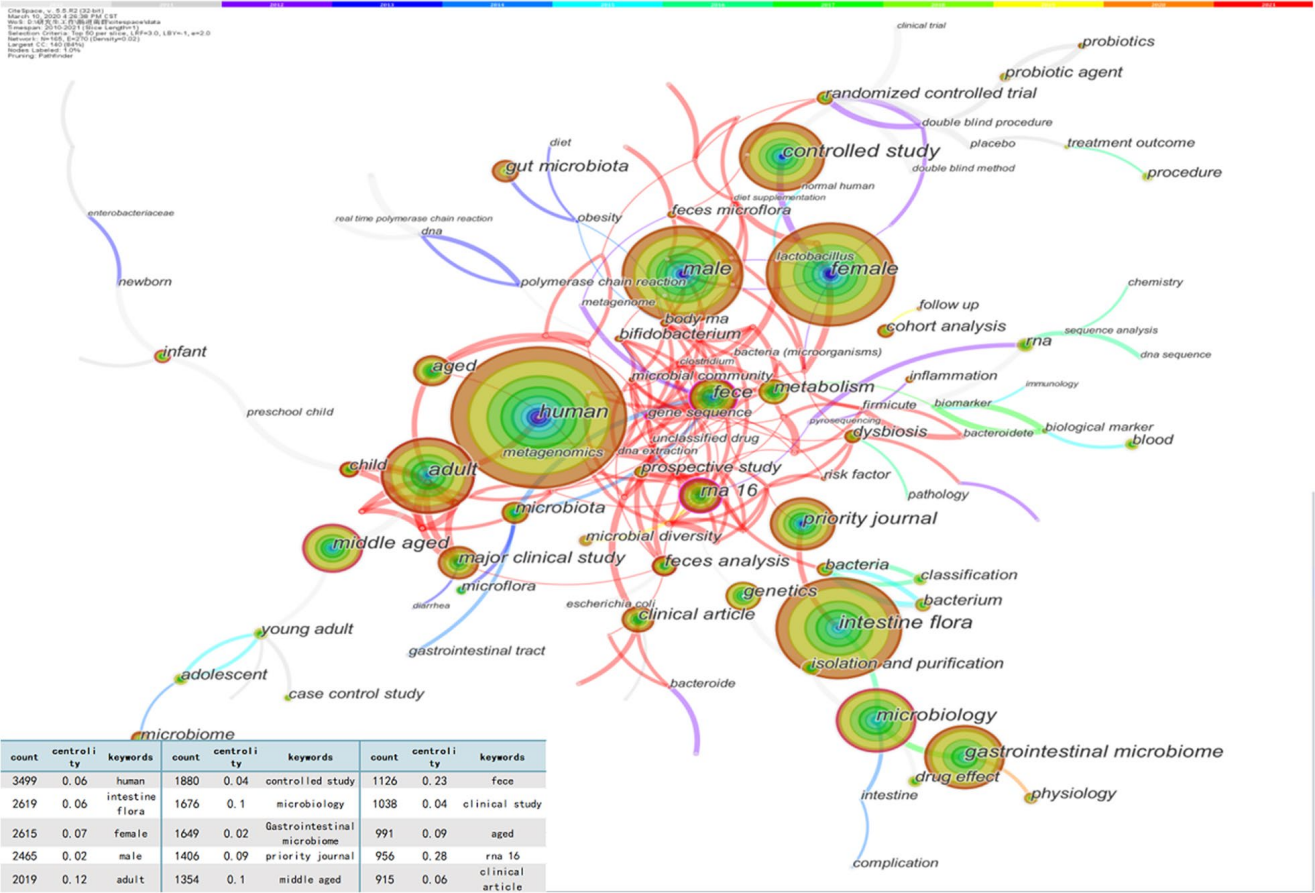
The Keyword co-occurrence knowledge map of the human gastrointestinal microbiome during 2010–2021. Each node represents a keyword, and the size of the node represents the frequency of their occurrence, and the line between nodes represents the intensity of co-occurrence, and the color of the line corresponds to the time range at the top of the picture. The frequency of keywords and centrality were listed is in the lower-left corner of the graph [28]

**Fig. 7:**
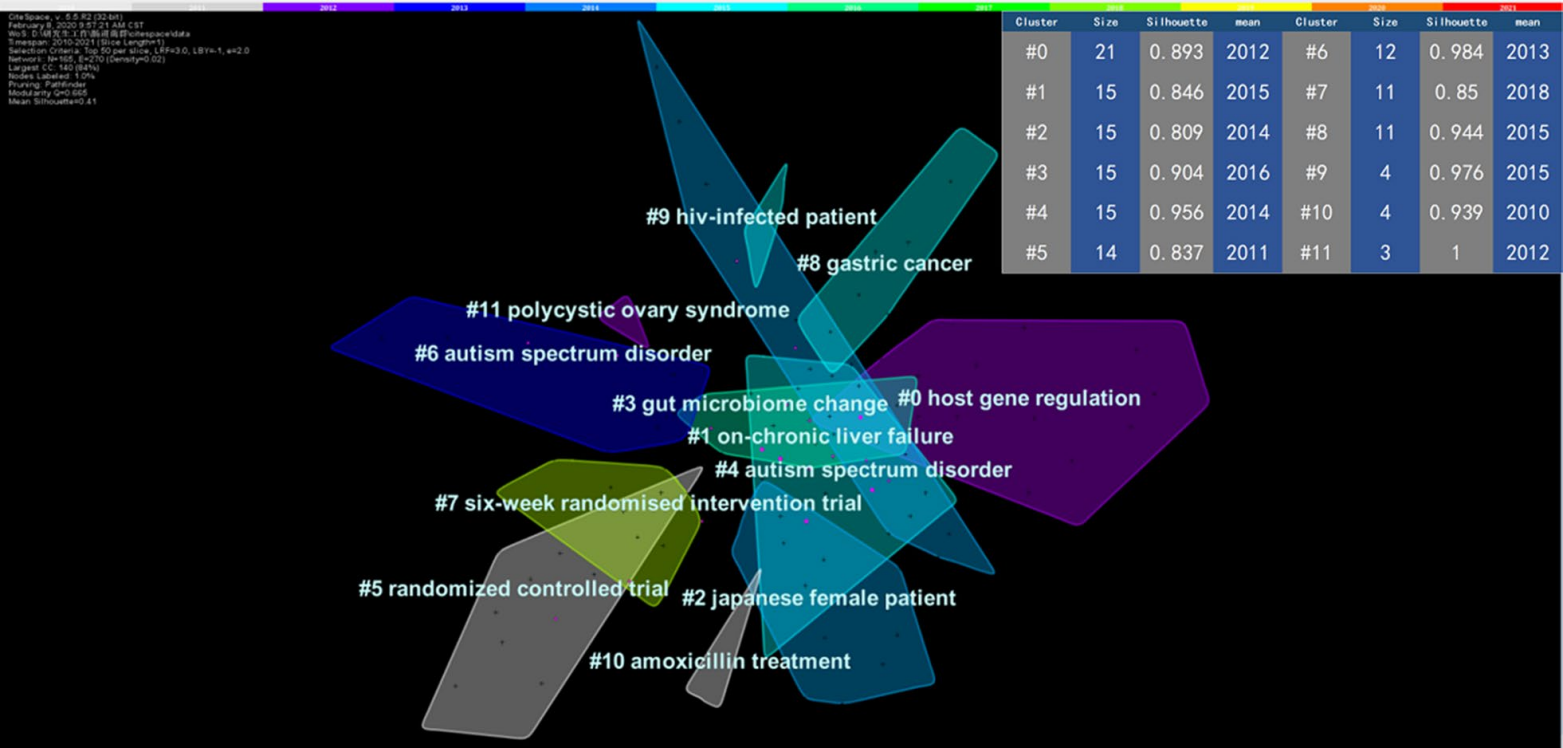
The Keyword clustering knowledge map of the human gastrointestinal microbiome during 2010–2021. CiteSpace uses Log-likelihood rate (LLR) to cluster closely related keywords. Different patterns represent a cluster. Tag # was assigned to the cluster, and the smaller the number is, the more keywords are in the cluster. The size of each cluster, the Silhouette value, and the mean publication year of articles in that cluster are shown in the upper right corner [28]

**Fig. 8:**
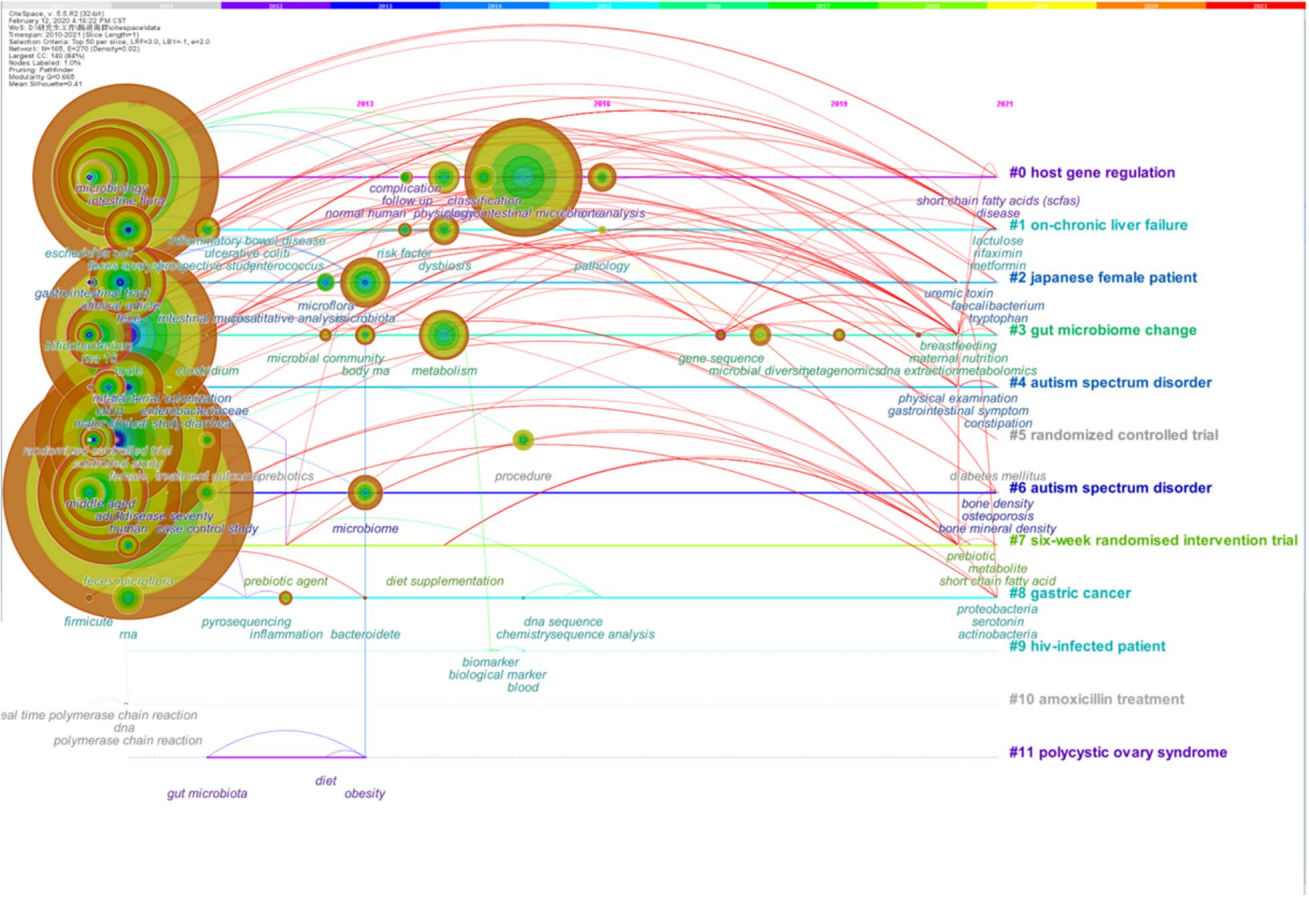
The timeline view of the human gastrointestinal microbiome during 2010–2021. In the timeline view, the keywords on the same horizontal line belong to the right cluster. The colors of lines and keywords in the view correspond to the colors of the time slice at the top [28]

**Fig. 9:**
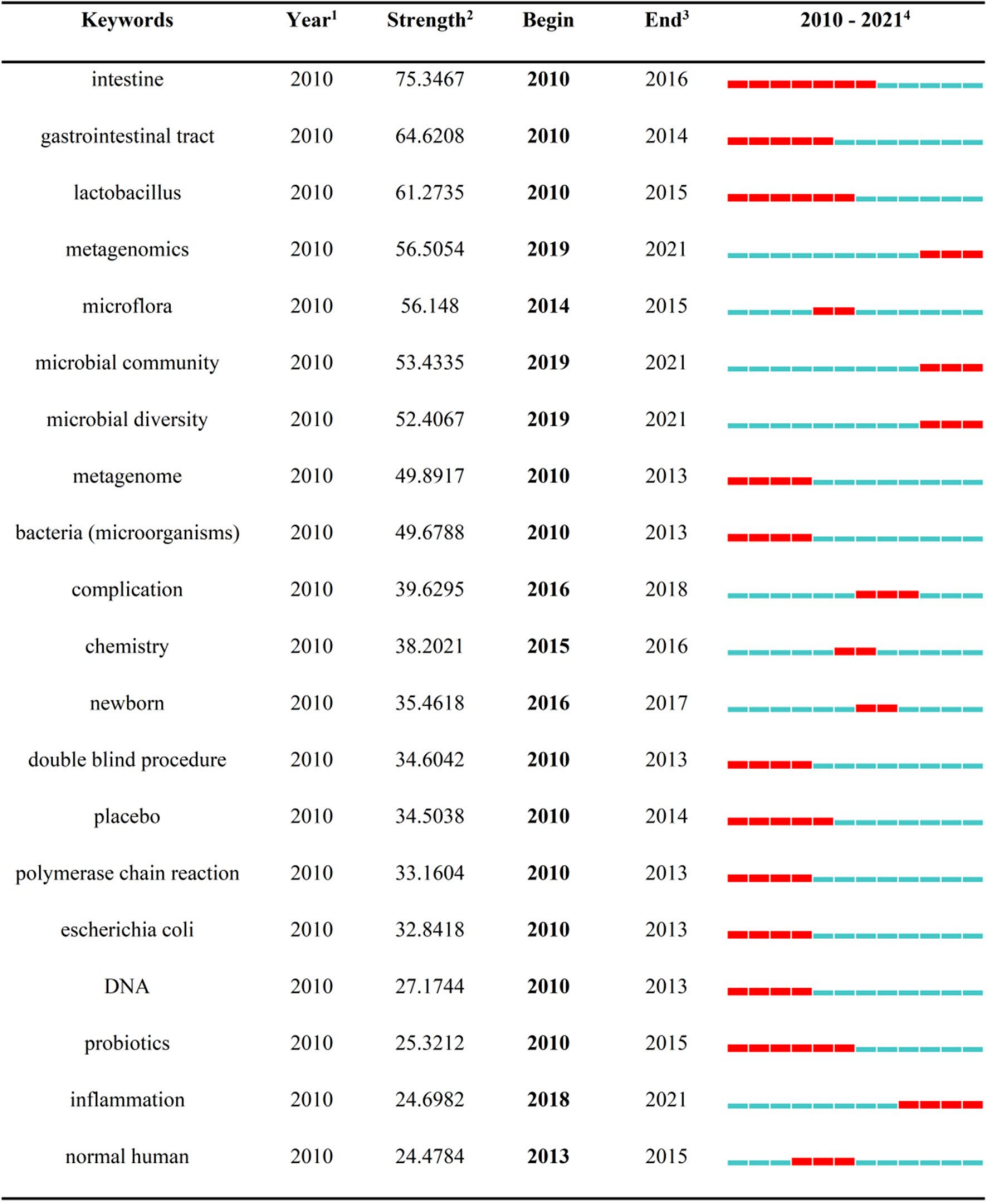
Top 20 Keywords with the Strongest Citation Bursts of the human gastrointestinal microbiome during 2010–2021. *1 The year in which this keyword first appeared. 2 the bursts’ strength of the keyword. 3 The year in which this keyword begins and ends the burst. 4 Red represents the period during which the keyword is burst

The articles were imported into CiteSpace, and the keywords were set as nodes. Through a series of software operations, the keyword co-occurrence knowledge map (Fig. 6), clustering knowledge map (Fig. 7), timeline view of keywords (Fig. 8), and keyword burst term map (Fig. 9) were obtained.

The keyword co-occurrence knowledge map (Fig. 6) contains 165 nodes and 270 lines, and the centrality is 0.02. The landmark nodes that are larger include human, intestinal flora, female, male, adult, and controlled study. They represent the most critical components of the human gastrointestinal microbiome field. The turning points with more connections include adults, microbiology, middle-aged, faecal, RNA 16, child, infant, and Bifidobacterium, indicating that they had higher centrality in the domain and were associated with more keywords.

There were 11 clustering patterns in the research field of the human gastrointestinal microbiome, which are shown in the keyword clustering knowledge map (Fig. 7). In addition, the top 20 most typical labels in each cluster are shown in Table 3.

**Table 3:** The most typical label in each cluster

Cluster	Label
#0	Host gene regulation; dynamic variation; single-center observational study; mild cognitive decline; formula-fed babies; term infant; covid-19 pandemic; faecal microbiota transplant service; microbiome-associated metabolite; gren syndrome; common pathogenic mechanism; kidney stone disease; functional difference; diagnostic model; metabolomic data; stool microbial extracellular vesicle; genomic profiling; intestinal t-cell receptor repertoire; cystic fibrosis; household well
#1	On-chronic liver failure; quantitative metagenomics; novo duplication; nuclear family; displaying neurodevelopmental disorder; fecal volatile compounds analysis; multi-center cohort study; preclinical detection; preterm infant; non-catheter related late-onset sepsis; gut-microbiome profile; food addiction; narrow spectrum; microbiota-dependent bile acid; anti-TNF therapy; untargeted metabolomics study; drug metabolism; enhanced characterization; large cross-sectional ibs cohort; intestinal diseases
#2	Japanese female patient; restricting-type anorexia nervosa; metabolomics profile; t1dm-onset pediatric patient; machine-learning algorithm; proinflammatory intestinal dysbiosis pattern; prospective study; shaping gut microbiota; rural africa; bronchial asthma; compound k; red ginseng; protopanaxadiol ginsenoside; healthy volunteer; preclinical study; beneficial anti-inflammatory effect; alcohol-associated liver disease; functional fingerprint; anti-TNF agent; interferon signature
#3	Gut microbiome change; sexual orientation; hiv infection; human gut archaeome; diverse haloarchaea; korean subject; systemic lupus erythematosus; glucocorticoid therapy; fecal microbiota signature; celiac disease patient; parkinsons disease patient; meconium microbiota share; vaginal microbiota; amniotic fluid microbiota; critical mutualism; competition interplay; sedentary lifestyle; normal-weight korean children; young obese; cross-sectional observational study
#4	Autism spectrum disorder; gut flora; lactobacillus strain; early childhood; oral antibiotics; preschool children; non-stunted children; potential cause; reduced gut microbial diversity; undernourished children; birth mode; gastrointestinal disorder; other diseases; dysbiosis signature; south african infant; colorectal cancer surgery; post-operative infectious complication; barrier function; double-blind study; perioperative probiotic treatment; altered gut microbial profile
#5	Randomized controlled trial; probiotic supplementation; gut functioning; secondary analyses; vegetable shot; human intervention study; vonoprazan-containing triple therapy; healthy human subject; globe artichoke; long-chain inulin; probiotic therapy; incipient arteriosclerosis initiate; follow-up study; perinatal probiotic intervention; infantile colic; lactobacillus reuteri dsm; lactobacillus salivarius cect5713; therapeutic efficacy; synbiotic supplementation; gastrointestinal comfort
#6	Autism spectrum disorder; randomized controlled trial; intestinal microbiota; metabolic health; probiotic supplementation; placebo-controlled study; gut microbiota dysbiosis; microbiotic surveillance; fatty acid; postprandial glucose control; probiotic formulation; tibetan patient; novel bacillus strain; human gut exert anticancer effect; malignancy type; nonalcoholic fatty liver disease; gut microbiota diversity; prebiotic effect; population-based cross-sectional study; multiple sclerosis correlate
#7	Six-week randomised intervention trial; omega-3 fatty acid supplementation; specific dietary fibre supplementation; chronic pancreatitis; caesarean section; crossover study; uk biobank; microbiota-derived short-chain fatty acid; bone health; genetic variation; dietary fiber; early life associate; prospective longitudinal infant cohort; specific gut microbiota signature; antibiotic resistant bacteria decolonization; integrative analysis; chinese patient; altered diversity; gut microbiota alteration; irritable bowel syndrome symptom
#8	Gastric cancer; probiotic strain bacillus subtilis; healthy microbiome; tryptophan pathway difference; current major depressive episode patient; severe tbi; community structure; states-veteran microbiome project study; fermentable oligosaccharide; dietary resistant starch type; intestinal microbiome disruption; infection prevention; microbiome disruption; long-term acute care hospital; breast milk jaundice; breastfed infant; microbiota characterization; blastocystis-free school-age children; dutch population
#9	HIV-infected patient; inflammatory bowel diseases; prognostic microbial biomarker; healthy middle-aged subject; randomised cross-over study; 3-d intervention; gut hormone; insulin sensitivity index; kernel-based product; gastrointestinal mucosa; spontaneous hiv controller; peripheral blood; intestinal microbiota correlate; bifidobacterium breve; mucosal-associated invariant t cell alteration; diabetic patient; combined antiretroviral therapy; lactobacillus population; metabolic interplay; new insight
#10	Amoxicillin treatment; bifidobacterium species; molecular characterisation; type ii diabete; microbial ecology; synbiotic food; metabolic profile; bacterial dna; helicobacter species; common gut; molecular analysis; mucosal bacterial communities; pediatric inflammatory bowel disease; intestinal microbiota; tetracycline resistance gene; probiotic lactobacillus reuteri; using 16 s sequence tag; pyrosequencing method; characterizing bacterial communities; faecal microbiota
#11	Polycystic ovary syndrome; population-based study; varied weight classification; cross-sectional comparison; fatty acid level; arabinoxylan oligosaccharide; metabolic marker; cross-over trial; fatty acid effect; overweight individual; intrinsic factor; early adolescent; shaping gut microbiota composition; viral dysbiosis; colon cancer development; obesity-related gut; fecal metabolomics; pubertal status; specific gut microbiota; intestinal tricarboxylic acid cycle intermediate; underweight status; healthy pre-obese subject

By combining the keyword clustering knowledge map (Fig. 7), timeline view (Fig. 8), and keyword burst map (Fig. 9), we found the evolutionary path of research hotspots. Amoxicillin treatment, vocabulary related to RCTs, the intestines, the gastrointestinal tract, lactobacillus, Escherichia coli, DNA, probiotics host gene regulation, and the metagenome began to attract attention in the early years (2010–2013). The middle stage (2014–2017) focused on microflora, complications, chemistry, newborns, normal humans, on-chronic liver failure, Japanese female patients, autism spectrum disorder, gastric cancer, and HIV-infected patients. In addition, in recent years (2018–2021), researchers have been interested in metagenomics, microbial communities, microbial diversity, inflammation, and other aspects.

## Discussion

The annual number of articles on the human gastrointestinal microbiome shows exponential growth (Fig. 1), indicating that this field is a research hotspot, and its popularity will continue to increase. This is consistent with previous studies [23, 39]. Researchers should give continuous attention to trends in related fields to uncover more connections between humans and the gastrointestinal microbiome. All countries and regions in the world have studied the human gastrointestinal microbiome (Fig. 2). The United States has the most publications, which may be related to the Human Microbiome Project (HMP) programme launched by the NIH in 2007 and the Gut Microbiota Brain AXIS programme in 2013 [40, 41]. The second most published country is China, which may be related to the importance attached to the study of the human microbiome mentioned by the National Natural Science Foundation of China, the 14th Five-Year National Key Research and Development Plan of the Ministry of Science and Technology and the Outline of the 2035 Vision Goals [42–44]. Although China is the second largest publishing country, there are no Chinese journals in the top 10 journals, which indicates that China can strengthen its construction of periodicals in this field. The largest collaborator is J Zhang's team from Shanghai Jiao Tong Univ, Sch Life Sci & Biotechnol in China, which focuses on probiotics and intestinal microorganisms. It is suggested that researchers from all countries continue to maintain close cooperation and share the latest research results on the human gastrointestinal microbiome.

The top 10 journals (Table 1) may be given priority when researchers publish and read articles on the human gastrointestinal microbiome because they have published a large number of studies on the human gastrointestinal flora. The reference co-citation knowledge map (Fig. 5) is clearly divided into three clusters according to time (from 2010 to 2013, from 2014 to 2018 and from 2019 to 2021), which indicates that the themes of each research stage are different. Researchers can read highly cited papers (those with large circle areas in Fig. 5) to find research hotspots at that stage. Moreover, beginning researchers can read the highly cited literature (Table 2) to help them understand the important findings in the field.

Compared with previous bibliometrics and visualized studies on the gastrointestinal microbiome that did not exclude animal studies, these studies on the human gastrointestinal microbiome mainly focus on the following aspects [23, 39]. As shown in the keyword co-occurrence knowledge map (Fig. 6), the larger landmark nodes can be divided into three categories: population, research methods, and detection methods. A large number of studies have been conducted on the relationship between age (newborn [45], infant [46], child [47], teenager [48], adult [49], middle aged [50] and aged [51]), gender (male [52] and female [53]) and population groups and gastrointestinal microorganisms. This may be related to researchers finding that the gastrointestinal floras of different populations are significantly different, which requires classification to further study the topic. Additionally, various research methods have been used to study this field. Experimental studies often include the effects of probiotics [54], faecal microbiota transplantation [55], Chinese medicine [56], and antibiotic therapy [57, 58] on the human gastrointestinal microbiome. Observational studies often include interactions between the gastrointestinal microbiome and various human diseases such as obesity [59], diabetes [60], and irritable bowel syndrome [61]. In addition, there are a variety of molecular biology technologies that have been used for gastrointestinal microbiome research. These technologies mainly include the following methods: bacterial culture technology based on molecular biotechnology, polymerase chain reaction (PCR), fluorescent in situ hybridization (FISH) [8], gene chips [62], and metagenome sequencing [63], and it is more popular to establish a gene bank of the gastrointestinal microbiome [64]. Each technology has advantages and disadvantages, and researchers can select the technologies suitable for their purposes.

As for research emphases, researchers’ exploration of the human gastrointestinal microbiome from 2010 to 2013 was at a relatively macro and superficial stage. Researchers have sought to determine how the gastrointestinal microbiome relates to humans. In 2011, one study combined 22 newly sequenced faecal metagenomes of individuals from four countries with previously published data sets to identify three robust clusters (referred to as enterotypes), which attracted intense attention at the time [32]. But since then, the discussion of enterotype has become less and less popular. Every year, a small number of studies look at the relationship between human enterotype and diet [33, 65], feces [66], human population [67], obesity [68], etc. Some researchers have suggested that grouping the microbiota of individual subjects into enterotypes, based on the dominance of certain genera may have oversimplified a complex situation [69]. Researchers have also explored the link between human gastrointestinal flora and certain diseases (such as type 2 diabetes, autism, obesity, irritable bowel syndrome, etc.) at this stage [70–72] and the relationship between Lactobacillus and human gastrointestinal flora [73–75]. In addition, a series of randomized controlled trials on the human gastrointestinal flora began to emerge at this stage [76–80]. The effect of antibiotics on human gastrointestinal flora was also a research hotspot during this period [81, 82].

From 2014 to 2017, increasingly more studies were conducted to determine the interaction between human gastrointestinal flora and various organs and systems (such as liver cirrhosis, Parkinson’s disease, rheumatoid arthritis, etc.) [83–85]. For instance, Francesco’s research suggested that the gut mycobiota contributed to the alteration of the intestinal microbial community structure in ASDs, which made it possible to develop new potential intervention strategies aimed at the relief of gastrointestinal symptoms in ASDs [86]. Dillon’s study suggested that an important relationship existed between altered mucosal bacterial communities and intestinal inflammation during chronic HIV-1 infection [87]. Moreover, the relationship between diet and human gastrointestinal flora is a research hotspot during this period [30, 88, 89].

From 2018 to 2021, researchers shifted their focus from certain types of gastrointestinal bacteria to the gastrointestinal microbial community [90] and microbial diversity [90]. Studies [91, 92] on the effects of the gastrointestinal microbiome and its metabolites on inflammation [93] and immunology [94] and their application as biomarkers [95] at the molecular level have also gradually become hotspots.

It is worth noting that researchers are often inspired by animal experiments to explore the mechanisms of diseases caused by the gastrointestinal microbiome and modify the gastrointestinal microbiome to treat disease; then, they judge the feasibility and safety of treatment methods [96]. However, human research has lagged behind animal models, and applying the results of animal experiments to humans requires more rigorous experiments and theories [18, 97]. At present, theories such as microbiota-gut-brain communication [98], gut-lung axis [99, 100], and enterohepatic circulation [101] can explain parts of the relationship between the gastrointestinal microbiome and various human organs and systems, but it is not yet completely clear. With the development of new technologies, such as omics and sequencing, the detection of the gastrointestinal microbiome has become more accurate. Big data also makes it possible to conduct comprehensive artificial intelligence research on multicentre, multidisease, and human gastrointestinal microbiome databases. Future research needs to be based on previous research results, combined with emerging technologies, and explore the relationship between gastrointestinal flora and humans at the molecular mechanism level to improve health.

There are some limitations to this study. In this study, only articles in the Scopus database were retrieved. Although using the Scopus database to conduct high-quality bibliometric analyses is widely accepted by researchers, it is still possible that some studies related to the human gastrointestinal microbiome have not been included, which may change the results of the study. This study examined only the last ten years of research on the human gastrointestinal microbiome, which may miss the development process of the human gastrointestinal microbiome from the start.

## Conclusion

In this study, 4444 original studies from January 2010 to February 2021 related to the human gastrointestinal microbiome were downloaded from the Scopus database and analyzed using CiteSpace to generate knowledge maps. The number of articles on the human gastrointestinal microbiome has increased rapidly in the past decade, and the scientific cooperation network showed that cooperation between different countries and institutions has been sufficient. The research topics focus on different populations, research methods, and detection methods. In addition, the research scope has gradually increased over time, and the research content has been gradually deeper and moving towards precision medicine. In short, the study of the human gastrointestinal microbiome is an ongoing research hotspot and contributes to human health.

## Data Availability

Not applicable.
